# Diverse macrophage populations contribute to distinct manifestations of human cutaneous graft-versus-host disease

**DOI:** 10.1093/bjd/ljad402

**Published:** 2023-11-27

**Authors:** Johanna Strobl, Laura M Gail, Laura Krecu, Shaista Madad, Lisa Kleissl, Luisa Unterluggauer, Anna Redl, Kveta Brazdilova, Simona Saluzzo, Philipp Wohlfarth, Hanna A Knaus, Margit Mitterbauer, Werner Rabitsch, Muzlifah Haniffa, Georg Stary

**Affiliations:** Department of Dermatology, Medical University of Vienna, 1090 Vienna, Austria; Wellcome Sanger Institute, Wellcome Genome Campus, Hinxton, Cambridge, UK; CeMM Research Center for Molecular Medicine of the Austrian Academy of Sciences, 1090 Vienna, Austria; Department of Dermatology, Medical University of Vienna, 1090 Vienna, Austria; CeMM Research Center for Molecular Medicine of the Austrian Academy of Sciences, 1090 Vienna, Austria; Department of Dermatology, Medical University of Vienna, 1090 Vienna, Austria; Wellcome Sanger Institute, Wellcome Genome Campus, Hinxton, Cambridge, UK; University of Cambridge, Cambridge, UK; Department of Dermatology, Medical University of Vienna, 1090 Vienna, Austria; CeMM Research Center for Molecular Medicine of the Austrian Academy of Sciences, 1090 Vienna, Austria; Department of Dermatology, Medical University of Vienna, 1090 Vienna, Austria; Department of Dermatology, Medical University of Vienna, 1090 Vienna, Austria; CeMM Research Center for Molecular Medicine of the Austrian Academy of Sciences, 1090 Vienna, Austria; Department of Dermatology, Medical University of Vienna, 1090 Vienna, Austria; CeMM Research Center for Molecular Medicine of the Austrian Academy of Sciences, 1090 Vienna, Austria; Department of Dermatology, Medical University of Vienna, 1090 Vienna, Austria; Department of Internal Medicine I, Bone Marrow Transplantation Unit, Medical University of Vienna, 1090 Vienna, Austria; Department of Internal Medicine I, Bone Marrow Transplantation Unit, Medical University of Vienna, 1090 Vienna, Austria; Department of Internal Medicine I, Bone Marrow Transplantation Unit, Medical University of Vienna, 1090 Vienna, Austria; Department of Internal Medicine I, Bone Marrow Transplantation Unit, Medical University of Vienna, 1090 Vienna, Austria; Wellcome Sanger Institute, Wellcome Genome Campus, Hinxton, Cambridge, UK; Department of Dermatology and NIHR Newcastle Biomedical Research Centre, Newcastle Hospitals NHS Foundation Trust, Newcastle upon Tyne, UK; Department of Dermatology, Medical University of Vienna, 1090 Vienna, Austria; CeMM Research Center for Molecular Medicine of the Austrian Academy of Sciences, 1090 Vienna, Austria

## Abstract

**Background:**

Graft-versus-host disease (GvHD) is a major life-threatening complication of allogeneic haematopoietic stem cell transplantation (HSCT), limiting the broad application of HSCT for haematological malignancies. Cutaneous GvHD is described as a post-transplant inflammatory reaction by skin-infiltrating donor T cells and remaining recipient tissue-resident memory T cells. Despite the major influence of lymphocytes on GvHD pathogenesis, the complex role of mononuclear phagocytes (MNPs) in tissues affected by GvHD is increasingly appreciated.

**Objectives:**

To characterize the identity, origin and functions of MNPs in patients with acute cutaneous GvHD.

**Methods:**

Using single-cell RNA sequencing and multiplex tissue immunofluorescence, we identified an increased abundance of MNPs in skin and blood from 36 patients with acute cutaneous GvHD. In cases of sex-mismatched transplantation, we used expression of X-linked genes to detect rapid tissue adaptation of newly recruited donor MNPs resulting in similar transcriptional states of host- and donor-derived macrophages within GvHD skin lesions.

**Results:**

We showed that cutaneous GvHD lesions harbour expanded CD163^+^ tissue-resident macrophage populations with anti-inflammatory and tissue-remodelling properties including interleukin-10 cytokine production. Cell–cell interaction analyses revealed putative signalling to strengthen regulatory T-cell responses. Notably, macrophage polarization in chronic cutaneous GvHD types was proinflammatory and drastically differed from acute GvHD, supporting the notion of distinct cellular players in different clinical GvHD subtypes.

**Conclusions:**

Overall, our data reveal a surprisingly dynamic role of MNPs after HSCT. Specific and time-resolved targeting to repolarize this cell subset may present a promising therapeutic strategy in combatting GvHD skin inflammation.

## Teaser

In acute graft-versus-host disease (GvHD), donor monocyte-derived macrophages rapidly enter the skin after haematopoietic stem cell transplantation and exhibit tissue-remodelling properties with expression of CD163, TGF-β and IL-10. In the upper dermis, macrophages are in close contact with lymphocytes and may induce a regulatory T-cell phenotype. In chronic cutaneous GvHD, skin macrophages are repolarized to CCR7^+^ macrophages producing proinflammatory cytokines (IFN-γ).

## Graphical Abstract

**Graphical Abstract:**
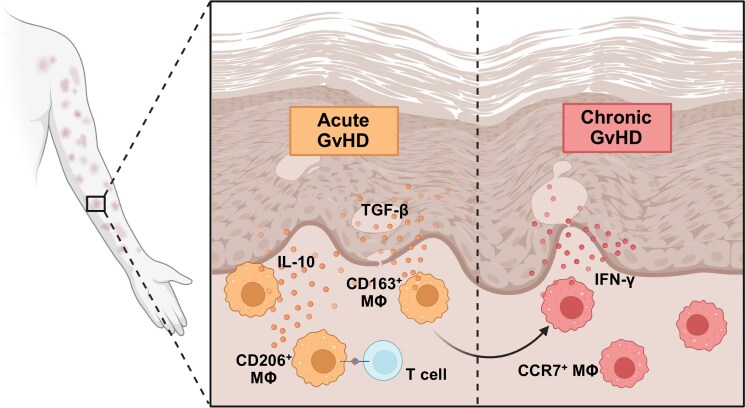

Plain language summary available online

Author Video: https://youtu.be/70PZxXtY-vA

Linked Article: Döbel *et al*. *Br J Dermatol* 2024; **190**:298.

**What is already known about this topic?**

- Cutaneous graft-versus-host disease (GvHD) is a life-threatening complication limiting the broad application of haematopoietic stem cell transplantation (HSCT).
- GvHD can manifest as acute- and chronic-type skin inflammation with distinct clinical presentations and lymphocyte cytokine ­profiles.
- Skin-infiltrating donor T cells significantly propagate GvHD, but little is known about donor and host macrophages (MΦs) as potential drivers of skin inflammation.

**What does this study add?**

- We detected donor- and host-derived MΦs as major cellular players in cutaneous GvHD clinical subtypes.
- Donor monocyte-derived MΦs rapidly entered the skin after HSCT and exhibited tissue-remodelling properties (CD163, transforming growth factor beta, interleukin-10 expression) in acute GvHD.
- In chronic cutaneous GvHD, skin MΦs had been repolarized to CCR7^+^ MΦs and produced proinflammatory cytokines (interferon gamma).

**What is the translational message?**

- Both host- and donor-derived MΦs were present in skin lesions and showed few transcriptomic differences, indicating rapid differentiation and polarization of donor mononuclear phagocytes to tissue MΦs as they enter the skin in acute GvHD.
- Furthermore, diverse MΦ responses in clinically distinct GvHD subtypes reflect the high plasticity of skin MΦs and illustrate the complex setting that is present in the cutaneous immune system after HSCT.
- Our results demonstrate a major role of MΦs as cellular players in GvHD-affected skin, with possibilities for therapeutic targeting.

##  

Cutaneous graft-versus-host disease (GvHD) is a major complication of allogeneic haematopoietic stem cell trans­plantation (HSCT), occurring in a large proportion of patients.^1^ Acute (a)GvHD of the skin is characterized by a potentially life-threatening lymphocytic inflammatory reaction mediated by host-derived tissue-resident memory T cells and donor lymphocytes infiltrating the skin.^2^ However, it is well established that both donor- and host-derived antigen-­presenting cells contribute to disease pathology.^3–6^

Tissue-resident macrophages (MΦs) are a major antigen-presenting cell subset in adult human barrier organs with a wide spectrum of pro- and anti-inflammatory properties, depending on their polarization state. MΦs have the ability to rapidly change their gene expression in response to environmental stimuli along a continuum of what has formerly been simplified as M1 and M2 polarization.

Skin-resident MΦs are induced by a T helper (Th)-2 cytokine environment^7^ and express surface receptors including CD11b, CD68 and CD206.^8^ Their detrimental role in inflammatory skin diseases has been shown for atopic dermatitis, psoriasis and discoid lupus skin lesions, where MΦs were characterized by the expression of *F13A1* or CD163 and genes related to chemotaxis and transforming growth factor beta (TGF-β) signalling.^9,10^ In a cytokine-rich environment, proinflammatory MΦs may produce interferon gamma (IFN-γ), which is a potent autocrine mediator inducing the MΦ phenotype found in psoriatic skin.^11^

However, skin-resident MΦs have also been appreciated for their anti-inflammatory properties and can promote tissue repair by local production of interleukin (IL)-10.^12^ Recently, a role for MΦs in GvHD pathology has been implicated in studies of tissues obtained from human HSCT recipients. Aasebo *et al*. detected a five-fold increase in donor-derived proinflammatory MΦs in colon biopsies of patients with gastrointestinal aGvHD.^13^ In cutaneous aGvHD, Jardine *et al*. found a population of monocyte-derived MΦs that mediated keratinocyte cytopathicity *ex vivo*.^6^

However, the functions and polarization profiles of donor- and host-derived MΦs in acute and chronic cutaneous GvHD remain unclear. Here, we used single-cell RNA sequencing (scRNA-seq) and tissue immunofluorescence (IF) in GvHD skin lesions from adult HSCT recipients to investigate MΦ subsets in acute and chronic cutaneous GvHD. We detected expanded CD163^+^ host and donor MΦs with low IFN-γ production and a tissue-remodelling cytokine signature (TGF-β, IL-10) in cutaneous aGvHD, and proinflammatory MΦs with decreased IL-10 production in cutaneous chronic GvHD.

## Materials and methods

### Patient inclusion, sampling and tissue processing

Thirty-six adult individuals presenting with acute, previously untreated cutaneous GvHD following allogeneic HSCT, at the University Hospital, Medical University of Vienna, Austria, were included in the study after obtaining appropriate fully informed written consent. GvHD type and stage were evaluated according to modified Glucksberg and National Institutes of Health consensus criteria.^14–17^ Of the 36 patients, 5 with previously untreated cutaneous aGvHD grade 2–3 were included for scRNA-seq, and 31 with previously untreated aGvHD, chronic lichenoid (cl)GvHD or chronic sclerotic (cs)GvHD were included for IF work-up. We performed 6-mm punch biopsies of lesional skin under local anaesthesia at disease onset before start of treatment with corticosteroids. Tissue was either deep frozen for IF work-up or immediately processed by enzymatic digestion for scRNA-seq. From patients presenting with aGvHD, blood was sampled in heparinized sample collection tubes and immediately processed for scRNA-seq. The study was approved by the local ethics committee, Medical University of Vienna (ECS 1087/2016).

### Cell sorting and library preparation

Single leucocyte suspensions were prepared from skin via enzymatic digestion and from whole blood using a Ficoll-Paque gradient, as previously described in detail.^18^ For sc-​RNA-seq using the 10X Genomics platform (10X Genomics, Pleasanton, CA, USA), CD45^+^ live cells were sorted into phosphate-buffered saline (PBS)/10% fetal calf serum for library preparation with the Chromium™ Single Cell 5′ Library & Gel Bead Kit v2 (10X Genomics) according to the manufacturer’s instructions. Briefly, sorted cells were partitioned into single-cell GEMs (Gel beads in EMulsion droplets) for cDNA synthesis and subsequently amplified and prepared for sequencing. The samples were sequenced at the Biomedical Sequencing Facility on the Illumina NovaSeq 6000 SP platform (Illumina, San Diego, CA, USA) in the 50-bp paired-end configuration. Raw sequencing data were processed as described below.

### Analysis of single-cell RNA-sequencing data

Raw sequencing data were quantified using the Cell Ranger single-cell gene expression software version 3.1.0 (10X Genomics) and aligned to the GRCh38 reference genome. Cells were filtered for detected gene number (< 200) and excessive mitochondrial gene expression (> 20%). Genes were filtered for expression in < 3 cells. After preprocessing, datasets were integrated and batch correction was performed using a BBKNN (batch balanced k nearest neighbours) graph-based data integration algorithm.^19^ Differential gene expression analysis was performed with the SCANPY toolkit^20^ using a Wilcoxon rank-sum test and Student’s *t*-test.

### Cell–cell interaction analysis

Intercellular communication between mononuclear phagocytes (MNPs) and T cells was inferred from single-cell transcriptome data using the R toolkit CellChat version 1.5.0.^21^ Based on cell clusters identified by the Leiden clustering algorithm, normalized count data were used to identify overexpressed genes and overexpressed interactions, and compute communication probability with the default package functions. Receptors/ligands were considered if expressed in > 15 cells per group.

### Tissue processing for multiplex immunofluorescence

For validation experiments, we collected lesional skin biopsies of 31 patients presenting with previously untreated cutaneous GvHD in different disease stages and pathologies: aGvHD (*n* = 15), clGvHD (*n* = 9) and csGvHD (*n* = 7). In addition, we collected excised skin from plastic surgery procedures of healthy individuals (*n* = 10). Tissue samples were embedded in Tissue-Tek^®^ optimum cutting temperature compound (O.C.T.™ Compound; Sakura Finetek, Torrance, CA, USA), deep frozen and cryosectioned. MΦs, cytokines and surface receptors were visualized with multistep IF staining. In brief, slides were defrosted, blocked with mouse serum (3%) and bovine serum albumin (2%) in PBS and incubated with directly conjugated primary antibodies overnight. CD68 ^+^ CD11b^+^ MΦs were costained either with anti-CCR7 or with anti-CD163. For cytokine labelling, cells contained in skin sections were previously permeabilized using Tween-20 solution. Primary anti-IFN-γ or anti-IL-10 antibody staining was performed by incubation for 2 h at room temperature, followed by secondary antibody labelling to increase signal strength. To reveal the cellular source of cytokines, MΦ labelling was performed on the same sections using incubation with anti-CD68 antibody overnight, followed by 4′,6-diamidino-2-phenylindole (DAPI) counterstaining. For visualization of MΦ–T-cell contact, IF was performed in aGvHD skin sections from three patients by incubation with directly conjugated anti-CD3, anti-CD68, anti-CD45 and anti-CD206 antibodies for 120 min at room temperature, and DAPI counterstaining after blocking steps as described above. Labelled sections were acquired using a ZEISS Axio Observer Z1 microscope (Zeiss Group, Oberkocken, Germany) with a TissueFAXS imaging system (TissueGnostics, Vienna, Austria) and processed using image analysis software (TissueGnostics).

## Results

### The mononuclear phagocyte fraction is expanded in graft-versus-host disease

**Figure 1:**
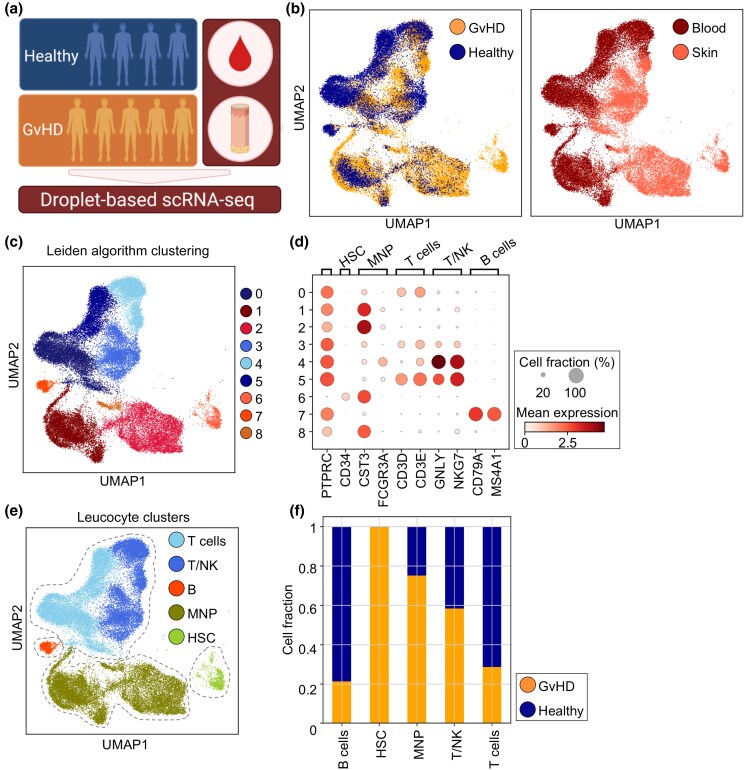
Cell populations in skin and blood of patients with acute graft-versus-host disease (aGvHD) and healthy donors. (a) Graphical overview of patient sampling. (b) UMAP clustering of aGvHD (*n* = 5) and healthy (*n* = 4) single-cell RNA-sequencing (scRNa-seq) datasets (left panel) and blood and skin cells according to cell barcode distribution (right panel). (c) UMAP showing Leiden clustering according to neighbourhood analysis in cells from (b). (d) Dot plot displaying cell type marker gene expression in Leiden clusters from (c). Data are shown as mean expression of respective genes in each group. (e) Leucocyte clusters identified by marker gene expression in (d). (f) Relative fraction of cell clusters identified in (e) derived from healthy (*n* = 4) and aGvHD (*n* = 5) datasets. Data are shown as mean cell fraction per cell type. HSC, haematopoietic stem cells; MNP, mononuclear phagocytes; NK, natural killer; UMAP, Uniform Manifold Approximation and Projection.

aGvHD of the skin may occur as early as post-transplant week 2, and is classically described as an adverse inflammatory reaction by skin-infiltrating donor T cells. However, it is well established that recovery of MNPs precedes lymphocyte engraftment after HSCT,^22,23^ prompting speculation on the contribution of MNP subsets to disease pathogenesis. To investigate MNP subsets in aGvHD, we performed scRNA-seq of blood and skin leucocytes from five HSCT recipients presenting with aGvHD of the skin (> grade 2) (Figure 1a, Table 1) and compared them with our previously published datasets from skin and blood of healthy ­individuals.^24^

**Table 1:** Clinical data of graft-versus-host disease (GvHD) patient donors (single-cell RNA-sequencing cohort)

	Sample 1	Sample 2	Sample 3	Sample 4	Sample 5
Age at HSCT	66 years	65 years	59 years	58 years	63 years
Sex	M	M	M	M	M
HSCT indication	MPN	AML	MPN	MDS	AML
Stem cell source	PB	PB	PB	PB	PB
GvHD onset (days)	50	90	20	47	21
Donor type	HLA-mismatched (9/10) URD	HLA-ident sibling	HLA-matched URD	HLA-matched URD	Haplo-ident daughter
Donor sex	Male	Female	Male	Male	Female
Conditioning	Flu/Treo30	Flu/Treo30	Flu/Treo30	Flu/Treo30	TBF

AML, acute myeloid leukaemia; Flu, fludarabine; HLA, human leucocyte antigen; HSCT, haematopoietic stem cell transplantation; ident, identical; MDS, myelodysplastic syndrome; MPN, myeloproliferative neoplasm; PB, peripheral blood; TBF, thiotepa, busulfan and fludarabine; Treo, treosulfan; URD, unrelated donor.

Following quality control filtering and doublet exclusion, we performed analysis on 24 346 sequenced skin- and 21 616 blood leucocytes [Figure 1b; Figure S1a (see Supporting Information)]. Following batch correction, Leiden clustering identified nine distinct cell states (Figure 1c). Differential expression of known cell-type marker genes allowed annotation of broad leucocyte clusters as T cells, cytotoxic T- and NK cells (T/NK), B cells, haematopoietic stem cells/immature leucocytes (HSCs) and MNPs (Figure 1d, e; Figure S1b). To identify the major leucocyte subsets contributing to inflammation in GvHD, we analysed the relative cell fraction for each cell type. We identified T/NK and MNP subsets to be relatively expanded in aGvHD compared with skin and blood from healthy donors (Figure 1f). Furthermore, immature leucocytes contained in the HSC cluster were derived almost exclusively from aGvHD datasets. When we mapped the relative fraction of cell types according to individual samples (Figure S1c), we noticed differential ratios of cells according to time of sampling after HSCT. Samples early after HSCT (< 3 weeks, GvHD3, GvHD5) contributed to MNP- and T-cell clusters, while B cells were mostly derived from healthy controls and samples > 40 days after HSCT (GvHD2, GvHD4).

### Acute graft-versus-host disease skin lesions harbour increased numbers of macrophages

**Figure 2:**
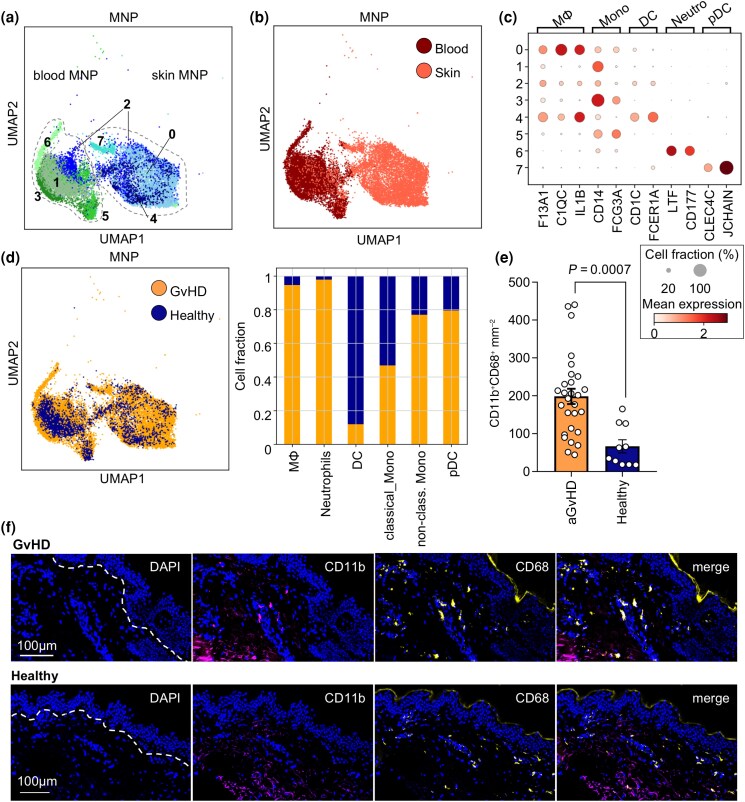
Expansion of macrophages (MΦ) in acute graft-versus-host disease (aGvHD) skin lesions. (a) UMAP showing Leiden subclusters from mononuclear phagocyte (MNP) cells from aGvHD (*n* = 5) and healthy control single-cell RNA-sequencing datasets (*n* = 4). (b) Distribution of skin and blood-derived cells in (a). (c) Dot plot showing marker genes used to identify MNP subclusters from (a). (d) Dataset origin in cells from (a). (e) Number of MΦ in aGvHD (*n* = 28) and healthy control skin (*n* = 10). Data are shown as CD11b/CD68 double-positive cells mm^–2^ ± SEM. Statistical analysis used an unpaired Student’s *t*-test. (f) Representative immunofluorescence staining of CD11b and CD68 in aGvHD and healthy control skin section. Dashed line indicates dermoepidermal border. Scale bar = 100 µm. DAPI, 4′,6-diamidino-2-phenylindole; DC, dendritic cells; Mono, monocytes; Neutro, neutrophils; p, plasmacytoid; UMAP, Uniform Manifold Approximation and Projection.

To further determine the role of MNPs in aGvHD, we next performed differential gene expression analysis of all aGvHD vs. healthy control datasets. Unsupervised analysis showed upregulation of MNP marker gene *CST3* and downregulation of T-cell-marker genes *CD3D* and *CD3E* in aGvHD (Figure S2a; see Supporting Information). Reclustering of cells identified as MNPs (Figure 2a) using the Leiden algorithm revealed eight distinct MNP transcriptional states (Figure 2a). MNPs clustered according to tissue origin in skin and blood MNPs (Figure 2b). We annotated distinct MNP clusters according to expression of published marker genes^9^ (Figure 2c; Figure S2b). Leiden clusters 1, 3 and 5 were almost exclusively derived from peripheral blood, and corresponded to classical and nonclassical monocytes by expression of *CD14* and *FCGR3A* (which encodes CD16). Cluster 6 contained circulating cells expressing neutrophil-related genes *LTF* and *CD177*, and was thus annotated as granulocyte cluster. Clusters 4 and 7 displayed a gene signature associated with dendritic cells (DCs) and were annotated as *CLEC4C*-expressing plasmacytoid DCs (pDCs, cluster *7*) and skin DCs, including Langerhans cells, expressing *FCER1A* and *CD1C* (cluster 4). Clusters 0 and 2 corresponded to tissue MΦs, characterized by the expression of *CD68*, *F13A1*, *C1QC* and *IL1B*.

To gauge the relevance of respective MNP subsets to GvHD inflammation, we compared the expression of pro- and anti-inflammatory marker genes in MΦ, monocyte and DC subclusters from GvHD samples (Figure S2c, top panels). Monocytes expressed high levels of *STAT1*, while *TGFB1* and *IL10RA* were expressed by DC and pDC subsets. Interestingly, cells of the MΦ cluster displayed the highest expression of *TNF*, *VEGFA* and *IL10* (Figure S2c, bottom panels), suggesting a tissue-remodelling cytokine signature.

We next mapped cell clusters back to their dataset origin and found the major fraction of MΦs, neutrophils, nonclassical monocytes and pDCs to derive from aGvHD datasets, while DCs were largely from healthy controls (Figure 2d). In GvHD dataset-derived MΦs, marker genes for cell proliferation (*MKI67*, *MYBL2*) and cell-cycle-regulated genes (*CCND1*) were upregulated (Figure S2d), suggesting *in situ* proliferation of cutaneous MΦ. To validate that the MΦ fraction was expanded in aGvHD skin, we investigated CD68 and CD11b protein expression in skin cryosections of individuals presenting with aGvHD > grade 2 (Figure 2e, f). We detected a mean number of 200 MΦs (CD68 ^+^ CD11b^+^ cells) mm^–2^ in aGvHD skin, which was significantly increased compared with healthy control skin. Taken together, our data show a clear expansion of tissue MΦs in aGvHD skin lesions.

### Activated conventional dendritic cells are present in acute graft-versus-host disease skin

Merely a small fraction of the DC cluster was derived from GvHD datasets (Figure 2d). While this finding is in line with delayed DC reconstitution after HSCT, we were interested in the phenotype of DC subsets present in aGvHD skin. To define DC subsets involved in GvHD, we subclustered cluster 4 (Figure 2a) using the Leiden algorithm. Within this DC cluster, we detected five distinct cell states (Figure S3a; see Supporting Information). Interestingly, only one subcluster (Leiden cluster 2) was mainly derived from GvHD skin datasets, the rest from healthy skin (Figure S3b). The GvHD DC cluster contained activated skin CD1C^+^ conventional DCs (*CD1C*, *FCER1A*, *CELC10A* expression) with high antigen-presenting function, indicated by increased expression of *NFKB1*, *CXCL8*, *BCL3* and *LGALS3* (Figure S3c). Functionally, Leiden cluster 2 resembled healthy skin-­derived DC subclusters with a similar expression of pro- and anti-inflammatory cytokines (Figure S3d).

### CD163^+^ tissue-remodelling macrophages are increased in acute graft-versus-host disease skin lesions

**Figure 3:**
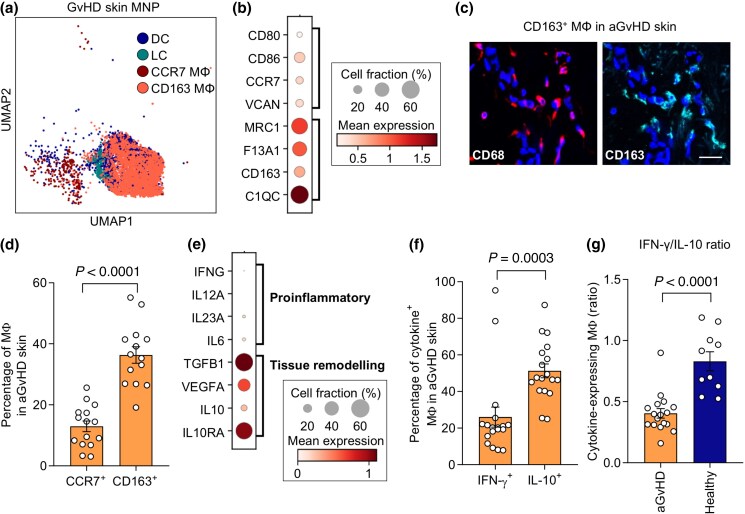
Acute graft-versus-host disease (aGvHD) skin lesions are marked by increased interleukin (IL)-10-producing CD163^+^ macrophages (MΦ). (a) UMAP clustering of aGvHD skin mononuclear phagocyte (MNP) cell states. (b) Dot plot of representative genes in cells from (a). (c) Representative image of CD68 and CD163 immunostaining in aGvHD skin. Scale bar = 20 µm. (d) CCR7^+^ and CD163^+^ MΦ in aGvHD skin (*n* = 15). Data are shown as percentage of MΦ (individual values and mean ± SEM). (e) Dot plot showing expression of profibrotic and proinflammatory cytokines and cytokine receptors in cells from (a). (f) interferon (IFN)-γ^+^ and IL-10^+^ MΦ in aGvHD skin (*n* = 18). Data are shown as mean percentage of cytokine-expressing MΦ (individual values and mean ± SEM). (g) Ratio of proinflammatory/profibrotic MΦs in aGvHD skin (*n* = 17) vs. healthy controls (*n* = 10). Data are shown as mean ratio of IFN-γ^+^ to IL-10^+^ MΦ. Bar represents mean ± SEM. Statistical analysis used paired (d, f) and unpaired (g) Student’s *t*-test. DC, dendritic cells; IFNG, IFN gamma; LC, Langerhans cells; TGFB, transforming growth factor beta; UMAP, Uniform Manifold Approximation and Projection.

To gain insights into the functional role of the numerically expanded MΦs in aGvHD skin lesions, we performed Leiden subclustering of MNPs derived from aGvHD skin lesions and retrieved four distinct cell states (Figure S2e). Annotation to known marker genes identified LC, DC and two MΦ clusters (Figure 3a; Figure S2f). We next investigated transcriptional profiles within the MΦ clusters isolated from aGvHD skin and detected high expression of genes described in skin-resident, anti-inflammatory, tissue-remodelling and tumour-associated MΦ, including *MRC1* (encoding CD206), *C1QC* and *CD163* (Figure 3b).^25,26^ Healthy skin MΦs displayed a similar transcriptome with low expression of pro-­inflammatory genes and migration marker CCR7 [Figure S4a (see Supporting Information)]. Interestingly, top differentially expressed genes between aGvHD and healthy skin MNPs included *MT2A* (encoding metallothionein-2), a gene upregulated in tissue repair MΦ,^27^  *TIMP1*, which regulates the angiogenetic capacity of MΦs^28^ and *PLIN2*, a gene involved in lipid storage, which is expressed by immunosuppressive tumour-associated MΦs (Figure S4b).^29^

To validate our findings, we investigated protein expression of scavenger receptor CD163 and the migration/lymph node homing receptor CCR7 on MΦs in aGvHD skin sections. In aGvHD, we detected significantly higher percentages of CD163^+^ MΦs compared with CCR7^+^ MΦs (Figure 3c, d). Accordingly, MΦs derived from aGvHD skin expressed tissue-remodelling cytokines *TGFB* and *IL10* on the RNA level (Figure 3e). Using intracellular cytokine staining on skin sections, we detected a significantly higher proportion of IL-10^+^ MΦs compared with IFN-γ^+^ MΦs in aGvHD (Figure 3f), which was less pronounced in healthy skin (Figure S4c–e). This resulted in an inverse ratio of proinflammatory vs. tissue-remodelling/anti-inflammatory MΦs in aGvHD (Figure 3 g).

### Macrophages interact with T cells in acute graft-versus-host disease skin

**Figure 4:**
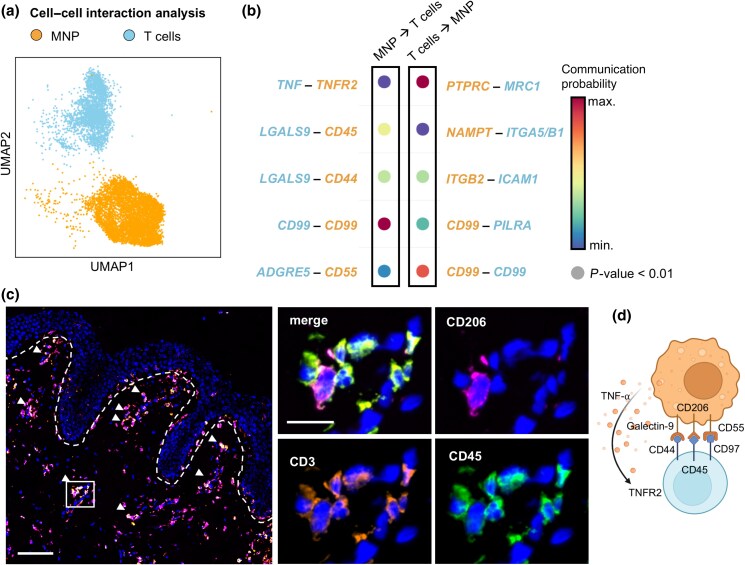
Interaction of macrophages (MΦ) and T cells in acute graft-versus-host disease (aGvHD) skin. (a) UMAP clustering of aGvHD skin-derived cells used for cell–cell interaction predictions. (b) Dot plot displaying putative receptor-ligand signalling of mononuclear phagocytes (MNP) to T cells (left) and T cells to MNP (right). Interactions were considered for *P*-values < 0.01 and significant gene upregulation in > 15 cells of each cluster. Data are shown as relative communication probability. (c) Image showing immunofluorescence labelling of CD68 ^+^ CD206^+^ and CD3 ^+^ CD45^+^ cells in aGvHD skin. Left panel is an overview image: arrows show cell–cell contact of MΦ and T cells, and line indicates dermoepidermal border (scale bar = 100 µm). Right panel shows magnification of MΦ–T-cell contact (scale bar = 20 µm). (d) Graphical representation of putative cell–cell interaction proteins between MΦ and T cells in GvHD skin. IFN-γ, interferon gamma; TNF-α, tumour necrosis factor alpha; TNFR, TNF receptor; UMAP, Uniform Manifold Approximation and Projection.

To address the consequence of anti-inflammatory MΦ polarization in aGvHD skin on T cells, we inferred putative cell–cell interactions from T-cell and MΦ scRNA states (Figure 4a) using the R toolkit CellChat.^21^ Unsupervised analysis yielded several significant receptor–ligand partners expressed by the investigated cell types (Figure 4b). Interestingly, the majority of signals predicted by CellChat were reported to induce regulatory T cells: (i) CD206 (encoded by *MRC1*) induces T-cell tolerance via inhibition of CD45 (*PTPRC*);^30^ (ii) the interaction of galectin-9 (*LGALS9*) with CD44 enhances the stability and function of adaptive regulatory T cells;^31^ and (iii) CD55 costimulation via CD97 (*ADGRE5*) induces IL-10 production in T cells.^32,33^ In addition, we detected potential instruction of tissue-regenerative T cells by MΦs via TNF/TNF receptor 2 (*TNFRSF1B*, alias *TNFR2*) signalling^34^ and interaction via *PILRA*, which has recently been reported to maintain CD8 T-cell quiescence in mice.^35^ To confirm a spatial relationship between MΦs and T cells for possible interaction, we performed immunofluorescence costaining in aGvHD skin and detected many instances of cell–cell surface contact (Figure 4c). In addition, we found CD206^+^ MΦs and CD45^+^ T cells in direct contact, indicating interaction via these receptors (Figure 4c, d). Overall, the polarization and functional profiles of cutaneous MΦs detected in our cohort suggest that MΦs induce a regulatory T-cell phenotype in the skin to limit GvHD inflammation.

### Remaining host skin-resident- and donor macrophages join forces in acute graft-versus-host disease skin

Although most tissue MΦ populations develop during embryogenesis from yolk sac progenitors, it is generally accepted that tissue-resident MΦs derived from HSC transplants repopulate the niche left by their naturally occurring counterparts^36^ and may even engraft as brain-resident MΦs.^37^ However, for skin-resident memory T cells we recently showed that host cells resist conditioning therapies preceding HSCT and remain in the skin for several years,^2^ where they may contribute to local GvHD and mediate Th2-driven inflammation at distant body sites.^38^

**Figure 5:**
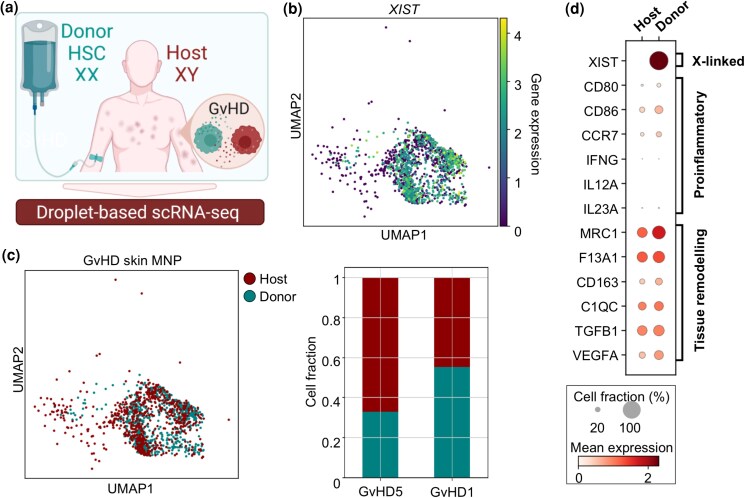
X-linked gene expression reveals similar transcriptional profiles of host and donor macrophages (MΦ). (a) Graphical representation of mismatched haematopoietic stem cell transplantation (HSCT) and sampling. (b) UMAP showing *XIST* gene expression in acute graft-versus-host disease (aGvHD) lesional skin MΦ of patients after mismatched HSCT (*n* = 2). (c) UMAP clustering (left panel) and relative cell fraction (right panel) of host and donor skin MΦ identified by *XIST* expression (b). (d) Dot plot showing proinflammatory and tissue-remodelling gene expression in cells from (c). IFNG, interferon gamma; IL, interleukin; scRNA-seq, single-cell RNA sequencing; TGFB, transforming growth factor beta; UMAP, Uniform Manifold Approximation and Projection.

To investigate the potential role of host tissue-resident MΦs in aGvHD skin inflammation, we analysed scRNA-seq datasets of two male individuals after sex-mismatched transplantation (Figure 5a). Notably, sex-mismatched HSCT is a well-established individual risk factor for the development of GvHD.^39,40^ Using the X-chromosome-linked gene *XIST* to distinguish donor from host cells,^41^ we identified 675 host- and 784 donor-derived cells within the MΦ skin cluster (Figure 5b). Unlike T cells,^2^ MΦs did not cluster according to genotype, indicating similar transcriptomic profiles of donor- and host-derived MΦs (Figure 5c, left panel). Interestingly, cell abundance analysis revealed relatively large fractions of remaining host MΦs in both aGvHD samples (Figure 5c, right panel). Lastly, gene expression analysis showed expression of tissue-remodelling genes by both donor- and host-derived MΦs (Figure 5d), and we found few genes with differential expression between *XIST*-negative host and *XIST*-expressing donor MΦs [Figure 4a–d; Figure S5a–d (see Supporting Information)]. In summary, we detected that host- and donor MΦs jointly populate GvHD skin lesions. Their similar transcriptional profiles indicate that engrafting donor MNPs rapidly adapt to tissue to become skin-resident MΦs.

### Diverse macrophage populations contribute to distinct manifestations of cutaneous graft-versus-host disease

Using disease onset and clinical presentation, cutaneous GvHD can be classified into an acute form and two chronic forms: aGvHD, clGvHD and csGvHD, respectively. These three most common clinical presentations of skin GvHD differ by histopathological features and distinct molecular signatures. Type-2 and Th-22 T-cell responses characterize aGvHD, while Th1/Th17 cytokines are upregulated in clGvHD, and increased transcripts of the type-1 cytokine IFN-γ were found in csGvHD.^42^

**Figure 6:**
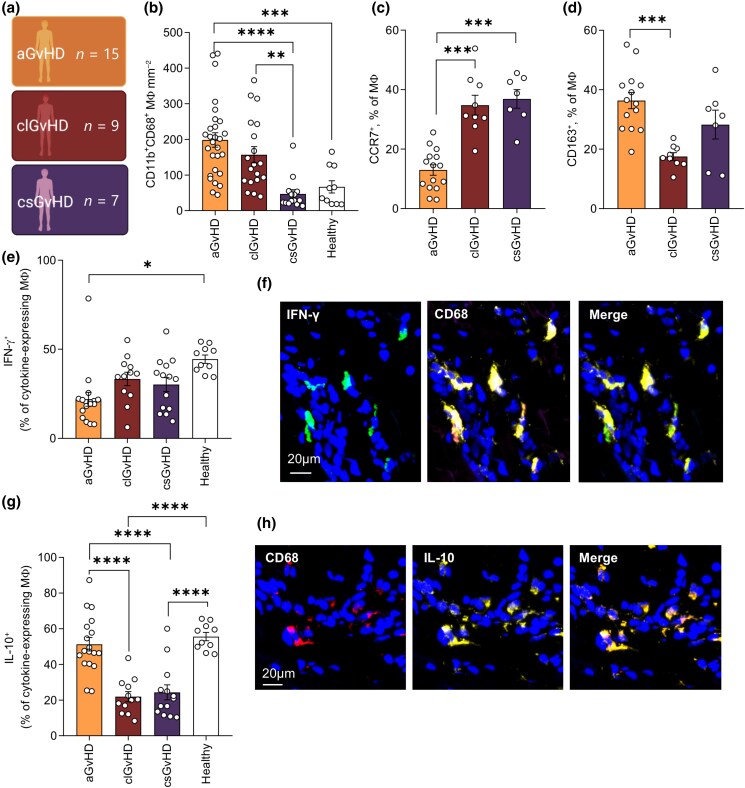
Distinct macrophage (MΦ) polarization profiles are found in acute graft-versus-host disease (aGvHD), chronic lichenoid (cl)GvHD and chronic sclerotic (cs)GvHD subtypes. (a) Graphical representation of GvHD patient sampling. (b) Quantification of CD11b ^+^ CD68^+^ cells in aGvHD (*n* = 15), clGvHD (*n* = 9) and csGvHD (*n* = 7) lesions vs. healthy skin (*n* = 10). Data are shown as number mm^–2^ ± SEM. (c, d) MΦ polarization in GvHD subtypes. Data are shown as percentage of MΦs positive for surface markers CCR7 (c) and CD163 (d). Bar indicates mean ± SEM. (e–h) Cytokine expression by CD68^+^ MΦ in samples from (b). Data are shown as mean ± SEM and individual percentage of interferon gamma (IFN-γ)^+^ (e) and interleukin (IL)-10^+^ cells among CD68^+^ MΦ positive for either cytokine. (f, h) Representative images of immunofluorescence staining for IFN-γ and IL-10 in MΦ clusters of the dermis of an aGvHD sample (scale bar = 20 µm). **P < 0.01, ***P < 0.001, ****P < 0.0001.

To investigate whether distinct MΦs may help shape these immunophenotypes, we investigated MΦ polarization profiles in a-, cl- and csGvHD skin lesions (Figure 6a). CD68 ^+^ CD11b^+^ MΦ numbers were increased in aGvHD and clGvHD, and decreased in csGvHD (Figure 6b). Interestingly, in contrast to the expanded CD163^+^ tissue-resident MΦs with tissue-remodelling and anti-inflammatory properties, we detected that aGvHD, clGvHD and csGvHD lesions were populated by CCR7^+^ MΦs (Figure 6c, d). Furthermore, chronic GvHD MΦs were characterized by normal IFN-γ and decreased IL-10 expression compared with aGvHD and healthy skin (Figure 6e–g). Overall, our findings support the notion that acute and chronic cutaneous GvHD subtypes indeed represent distinct adverse immunological reactions after HSCT.

## Discussion

In the present study we used scRNA-seq and tissue IF to resolve the cellular heterogeneity of MNPs in cutaneous GvHD. Our data show a significant expansion of both host- and donor-derived MΦs in aGvHD skin and a clear distinction from chronic GvHD MΦ polarization, indicated by transcriptional profile, CD163 surface protein expression, *TGFB* transcription and IL-10 cytokine production.

Overall, the importance of myeloid cells in the initiation and propagation of GvHD has been widely acknowledged.^4^ A histopathological study investigating aGvHD skin lesions found that increased CD163^+^ cell infiltration was a significant predictive factor for steroid-refractory GvHD as well as a negative prognostic factor for survival.^43^ However, more recent studies suggest a protective role of this type of MΦ in murine GvHD and radiation injury, where mesenchymal stem cell-educated CD163^+^ MΦs improved survival.^44^ In our study, we corroborate the presence of increased numbers of CD163^+^ cells in aGvHD skin and characterized them as tissue-remodelling CD11b ^+^ CD68^+^ MΦs with increased production of the anti-inflammatory cytokine IL-10 and potential instruction of regulatory T cells.

This type of MΦ polarization has previously been found in other, more common inflammatory skin diseases including atopic dermatitis and psoriasis, which both share some clinical and molecular features with aGvHD.^9^ Although skin-resident MΦs may produce high levels of IL-10 and TGF-β, providing anti-inflammatory functions in tissue repair, they are highly plastic, and promote tissue inflammation in Th-2-mediated conditions including contact dermatitis and asthma,^45,46^ rather than dampening the inflammatory response. Interestingly, TGF-β is a well-characterized factor promoting establishment of tissue-resident memory T cells with cytotoxic functions in human skin.^47^ Therefore, while our data generally suggest an anti-inflammatory role of MΦs in previously untreated aGvHD, changes in the skin environment could lead to rapid repolarization in the later stages of disease, including steroid-refractory GvHD. Notably, therapeutic repolarization of MΦs – from anti- to proinflammatory – has been implicated in preclinical models as a potential treatment of solid tumours.^48^ Conversely, specific targeting to increase anti-inflammatory functions of the numerous MΦs in GvHD skin may present a novel therapeutic strategy to induce regulatory T cells after transplantation.

After HSCT, immunosuppressive therapy dampens inflammatory responses induced by loss of epithelial integrity, microbial dysbiosis and host-cell antigen presentation.^49,50^ Both host and donor antigen-presenting cells were shown to play a prominent role in GvHD tissue inflammation.^6,51,52^ In patients after sex-mismatched transplantation, we found host- and donor-derived MΦs to exhibit highly similar transcriptional profiles in GvHD-affected tissues. This is surprising, as tissue-resident MΦs differ from monocyte-derived macrophages by their bone marrow origin, and fulfil niche-specific homeostatic functions.^8^ However, MΦs are remarkably plastic immune cells^53^ and the cytokine milieu present in GvHD-affected tissues may prompt incoming donor monocyte-derived MΦs to rapidly adapt and polarize towards a skin-resident phenotype.

In line with the diverse T-cell responses present in cutaneous GvHD subtypes,^42^ we found MΦ responses in aGvHD to be distinct from chronic GvHD lesions, where we detected MΦs with low IL-10 production, high CCR7 expression and relatively higher IFN-γ production. Our data are compatible with findings of a recent study investigating chronic GvHD skin using bulk RNA sequencing, which described a cytokine signature dominated by IFN-γ in lichenoid and sclerotic skin lesions.^54^ Furthermore, clGvHD was associated with increased expression of *TREM1*, a molecule that characterizes proinflammatory MΦs in inflammatory bowel disease.^55^ In an IL-17-dependent murine model of scleroderma-like GvHD, donor MΦs expressed CD206 and TGF-β but not iNOS, identifying them as profibrotic skin-resident macrophages,^52^ which suggests central differences between murine cutaneous GvHD models and human skin.

Overall, contrasting MNP- and T-cell phenotypes in acute and chronic GvHD potentially reflects differences in disease pathogenesis and pathophysiology: aGvHD develops upon barrier dysfunction and dysbiosis after conditioning therapy,^56^ and the subsequent IL-4-containing cytokine milieu^57^ may induce skin homing of donor monocyte-derived MΦs with tissue-remodelling properties. IL-1 and IL-6-rich environments in damaged skin later after HSCT may propagate the proinflammatory MΦ bias in chronic GvHD. Finally, the lack of anti-inflammatory MΦs – for example as a long-lasting effect of total body irradiation^58^ – may contribute to sclerosis in csGvHD.

In summary, our results demonstrate a major role of MΦs as cellular players in GvHD-affected skin, with possibilities for therapeutic targeting. In our study, both host- and donor-derived MΦs were present in skin lesions and showed few transcriptomic differences, indicating rapid differentiation and polarization of donor MNPs to tissue MΦs as they enter the skin in aGvHD. Furthermore, diverse MΦ responses in clinically distinct GvHD subtypes reflect the high plasticity of skin MΦs and illustrate the complex setting that is present in the cutaneous immune system after HSCT.

## Supplementary Material

ljad402_Supplementary_Data

## Data Availability

Graft-versus-host disease single-cell RNA-seq datasets related to this article are deposited via NCBI’s Gene Expression Omnibus (GEO)^59^ in the form of deidentified count matrices, accessible through GEO series accession number GSE236264. Previously published healthy control datasets are available via GEO series accession number GSE184320.
