# Supplementary material for: Diverse macrophage populations contribute to distinct manifestations of human cutaneous graft-versus-host disease
Source: Br J Dermatol. 2023 Nov 27;190(3):402–14. doi: 10.1093/bjd/ljad402 (PMC10873647; doi:10.1093/bjd/ljad402)
Supplement: ljad402_Supplementary_Data [file ljad402_supplementary_data.zip › ljad402_Supplementary_Data.docx]

**Supporting Information**

**Appendix S1 Supplementary Figures**

**
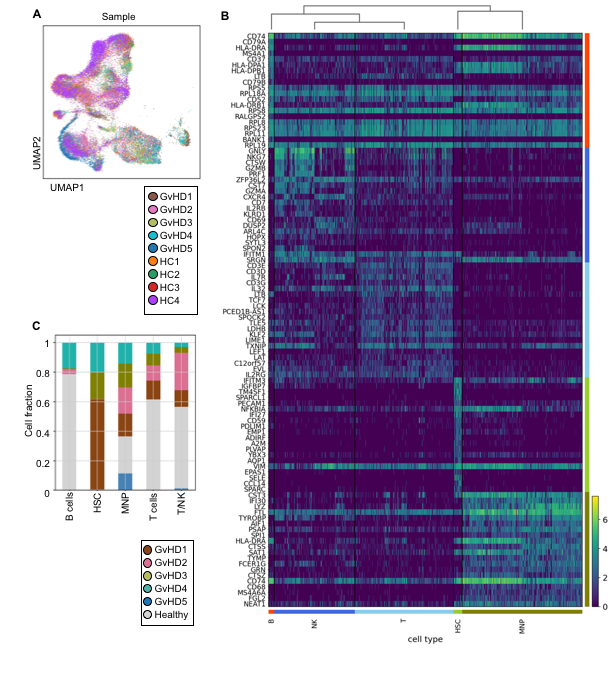
 Figure S1. (**A) UMAP clustering of GvHD and healthy scRNA-seq datasets, colors indicate individual donors. (B) Heatmap showing differentially expressed genes in leukocyte clusters identified in Figure 1D. Color indicates RNA expression; differential expression was determined using t-test (n=9: aGvHD n=5, healthy n=4). (C) Relative fraction of cell types derived from individual aGvHD samples and healthy control dataset. Data shown as mean cell fraction per cell type.

**
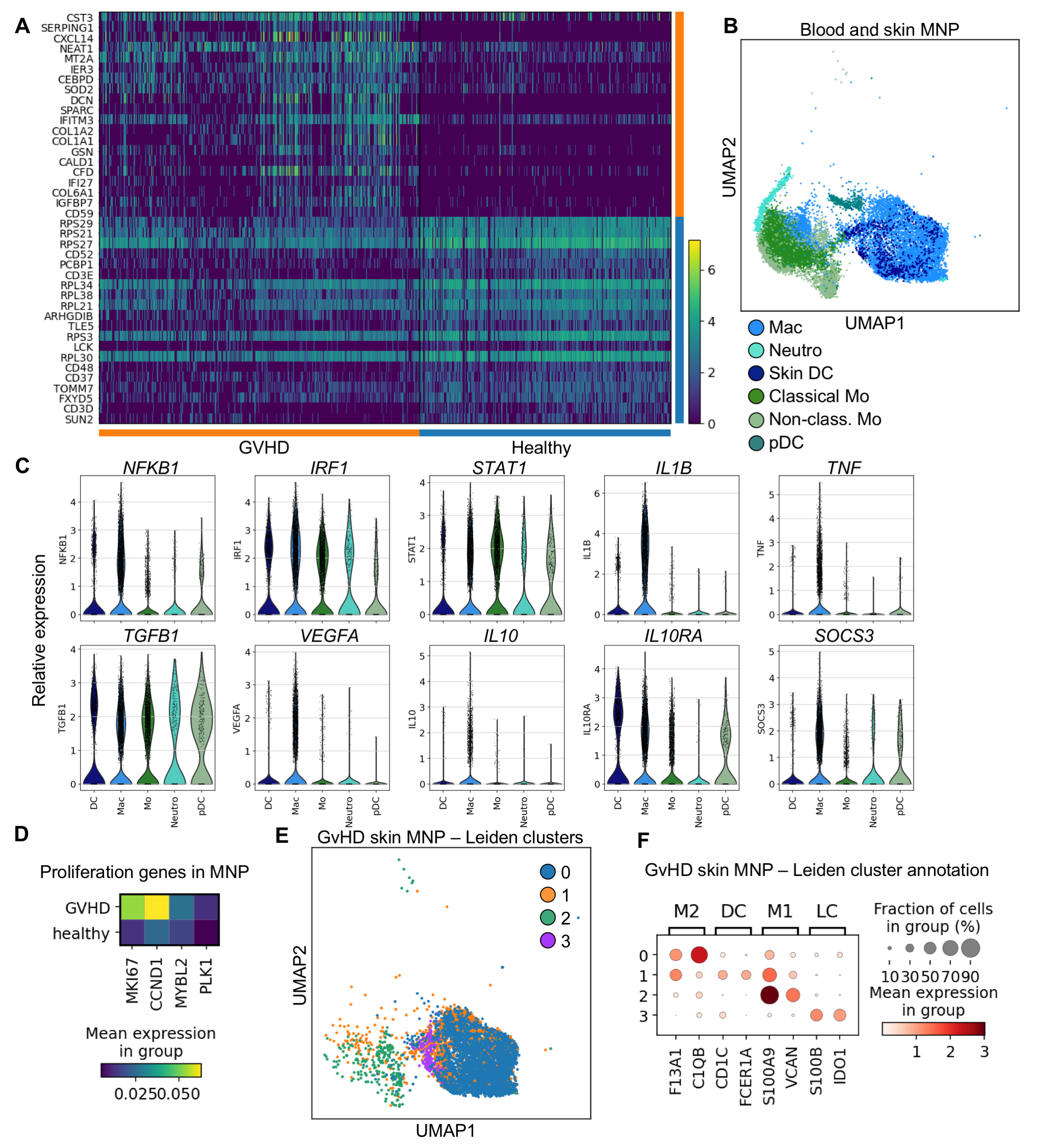
**

**Figure S2.** (A) Heat map showing top differentially expressed genes between GvHD (n=5) and healthy (n=4) scRNA-seq datasets (all cells). Statistical analysis using t-test. (B) UMAP showing MNP sub-clusters as identified by common marker genes (n=9). (C) Violin plots showing relative expression of pro-inflammatory (top panels) and anti-inflammatory/tissue remodeling (bottom panels) marker genes in cell state clusters from (B), from GVHD samples only (n=5). (D) Heat map of genes associated with cell proliferation, data shown as mean expression in GvHD skin and healthy skin MNP. (E) UMAP of Leiden clusters of GvHD skin-derived MNP subsetted from (B) (n=5). (F) Dot plot showing marker genes for annotation of Leiden clusters in (E).

**
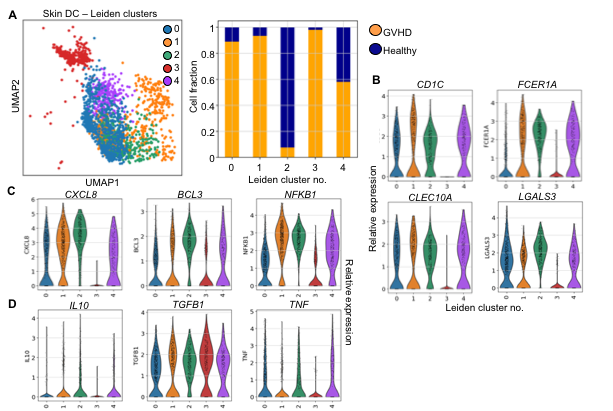
**

**Figure S3.** (A) UMAP of distinct cell states of skin-derived DC (subsetted from Figure 2A) as identified by Leiden clustering (left panel) and relative fraction of Leiden clusters derived from healthy (n=4) and aGvHD (n=5) datasets (right panel). Data shown as mean cell fraction per cell type. (B-D) Violin plots showing relative expression of conventional DC marker genes (B), activation marker genes (C) and cytokine genes (D) in cells from (A).

**
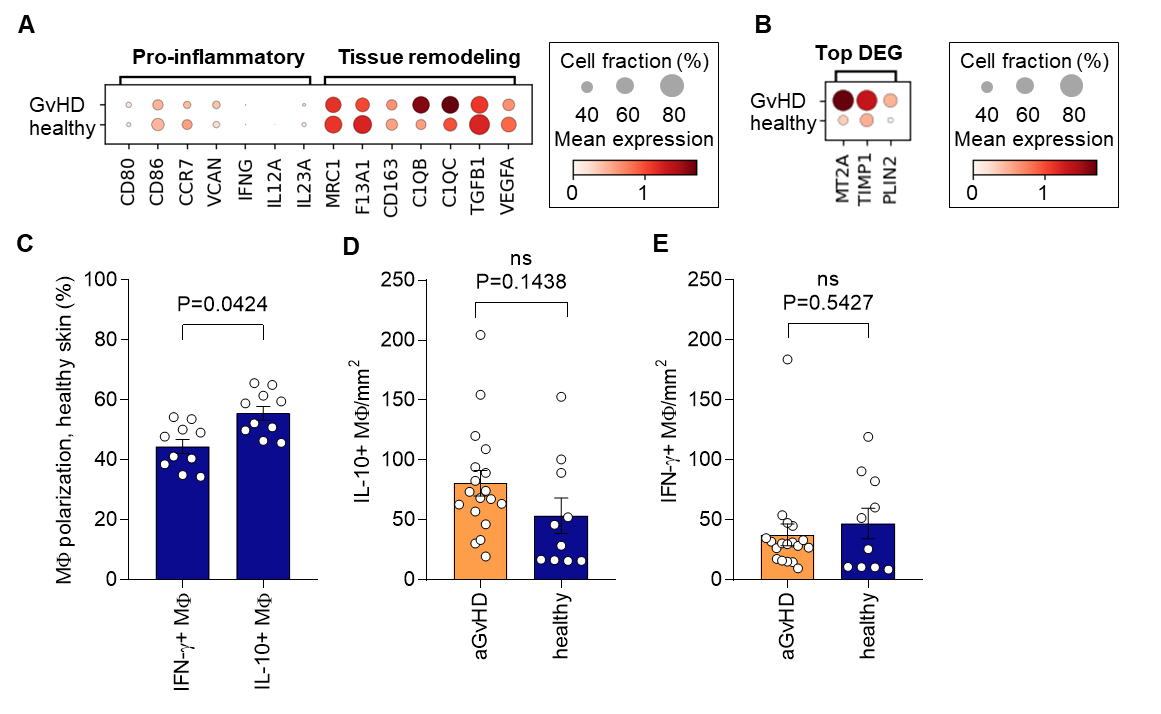
**

**Figure S4. GvHD lesional MΦ exhibit a polarization profile similar to healthy control skin.** (A-B) Dot plot showing expression of common MΦ marker genes (A) and top differentially expressed genes (B) in MNP subclusters of aGvHD (n=5) and healthy (n=4) scRNA-seq datasets. (C) Distribution of cytokine-expressing MΦ in healthy control skin. Data shown as percentage of IFN-γ+ and IL-10+ cells among all cytokine-expressing MΦ detected by IF staining, n=10. D-E) IL-10+ (D) and IFN-γ+ (E) MΦ in aGvHD (n=18) vs. healthy skin (n=10). Data shown as IL10+ and IFN-γ+ CD68+ cells/mm^2^ +/- SEM as detected by IF staining of skin cryosections. Statistical analysis using paired (C) and unpaired t-test (D, E).

**
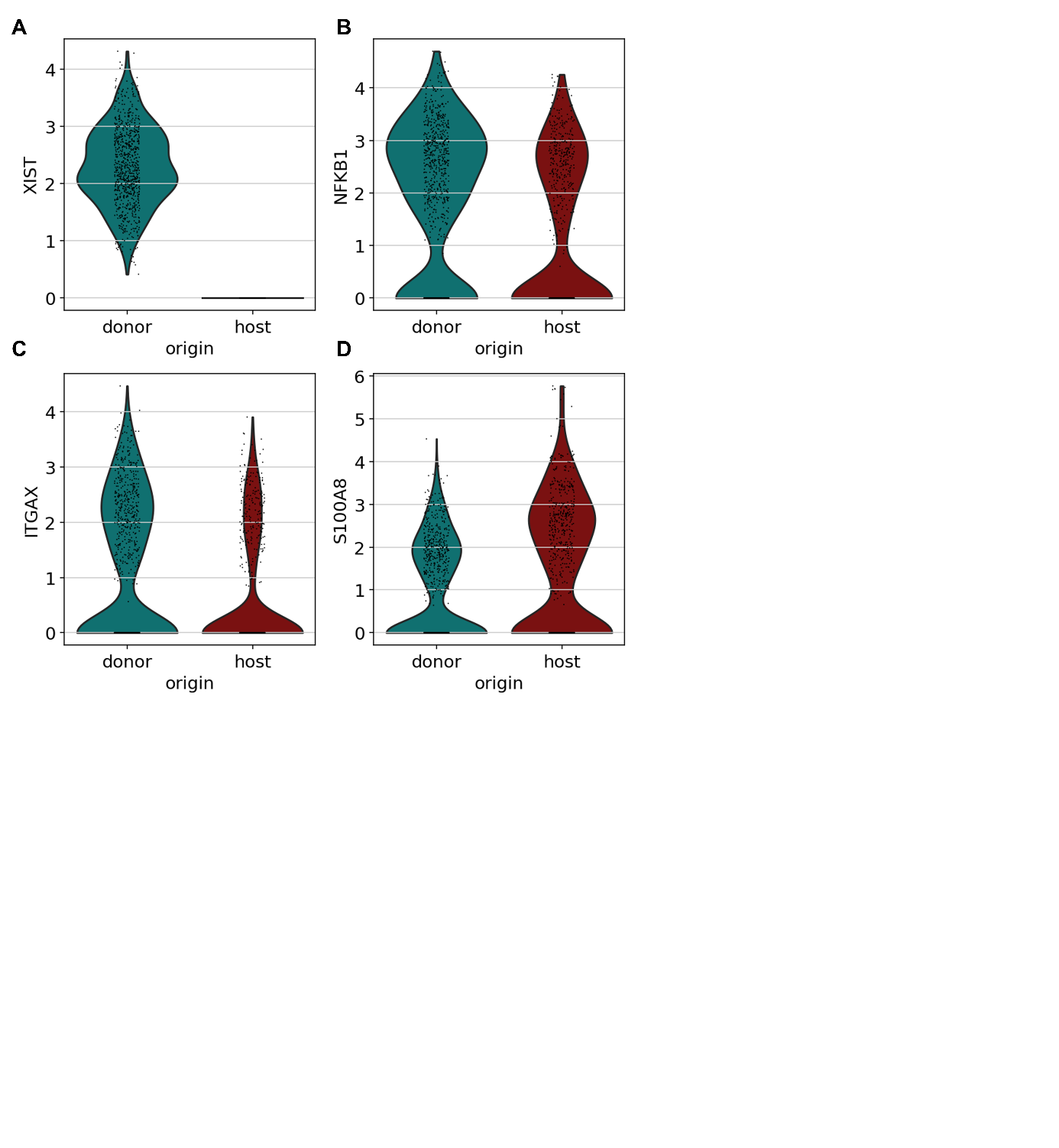

Figure S5. Differential gene expression in host and donor MΦ.** Violin plots of cells expressing *XIST* (A), *NFKB1* (B), *ITGAX* (C) and *S100AB* (D) in host and donor MΦ. Data shown as expression per cell and range. Selected genes are among the top 20 DEG between host and donor MΦ.
